# Shedding of neurexin 3β ectodomain by ADAM10 releases a soluble fragment that affects the development of newborn neurons

**DOI:** 10.1038/srep39310

**Published:** 2016-12-19

**Authors:** Erika Borcel, Magda Palczynska, Marine Krzisch, Mitko Dimitrov, Giorgio Ulrich, Nicolas Toni, Patrick C. Fraering

**Affiliations:** 1 Brain Mind Institute and School of Life Sciences, Ecole Polytechnique Fédérale de Lausanne (EPFL), CH1015 Lausanne, Switzerland; 2 Department of Fundamental Neurosciences, University of Lausanne (UNIL), CH1015 Lausanne, Switzerland; 3 Foundation Eclosion, CH1228 Plan-Les-Ouates & Campus Biotech Innovation Park, CH1202 Geneva, Switzerland

## Abstract

Neurexins are transmembrane synaptic cell adhesion molecules involved in the development and maturation of neuronal synapses. In the present study, we report that Nrxn3β is processed by the metalloproteases ADAM10, ADAM17, and by the intramembrane-cleaving protease γ-secretase, producing secreted neurexin3β (sNrxn3β) and a single intracellular domain (Nrxn3β-ICD). We further completed the full characterization of the sites at which Nrxn3β is processed by these proteases. Supporting the physiological relevance of the Nrxn3β processing, we demonstrate *in vivo* a significant effect of the secreted shedding product sNrxn3β on the morphological development of adult newborn neurons in the mouse hippocampus. We show that sNrxn3β produced by the cells of the dentate gyrus increases the spine density of newborn neurons whereas sNrxn3β produced by the newborn neuron itself affects the number of its mossy fiber terminal extensions. These results support a pivotal role of sNrxn3β in plasticity and network remodeling during neuronal development.

Neurexins (Nrxns) are cell adhesion molecules involved in synaptic activity, maturation and maintenance through the recruitment of pre- and postsynaptic proteins and receptors^1^,^2^. Mutations in Nrxn isoforms have been associated with neurodevelopmental disorders including autism spectrum disorders (ADS) and schizophrenia, both of which are characterized by an altered ratio of excitatory to inhibitory brain transmission^1^,^3^,^4^,^5^,^6^. Moreover, synaptic dysfunction in Alzheimer’s disease (AD) is likely to involve alterations in synaptic cell adhesion molecules processing, including Nrxn and neuroligin (NL)^7^,^8^,^9^. Supporting this observation, a recent meta-analysis study showed that the gene Nrxn3 might be related to susceptibility to AD^7^. The precise functional implications of these proteolytic processes and mutations in Nrxn may be further investigated in order to develop new potential therapies.

We and others have reported a neuronal activity-dependent proteolytic processing of both Nrxn3β and its main partner - neuroligin1 (NL1) - resulting in the generation of two types of cleavage products: (i) the N-terminal soluble and secreted ectodomains sNrxn3β and sNL1, and (ii) the intracellular domains Nrxn3β-ICD and NL1-ICD^8^,^10^,^11^,^12^. Following their shedding by metalloproteses, both Nrxn3β and NL1 C-terminal fragments (CTFs) are cleaved by γ-secretase, an intramembrane-cleaving protease that controls a still growing list of cell functions^13^, resulting in the generation of ICDs and P/Aβ-like peptides. Recent studies suggest that the physiological role of metalloproteases/γ-secretase enzymes includes regulation of axonal growth as well as synapse remodeling and maintenance^5^,^14^. In fact, it has been reported that the neurexin-ICD fragment can translocate into the nucleus and regulate gene expression through the activation of signaling pathways involved in important functions like cell fate, adhesion, migration or synaptogenesis^15^. Despite this progress, the potential synaptic functions of Nrxn processing and its cleavage products are largely unknown.

In the present study we identified for the first time the precise sites at wich Nrnx3β is processed as well as the specific enzymes involved in the processing. We show that Nrxn3β is processed by ADAM10 and ADAM17 at conserved sites in the extracellular part of the sequence to produce soluble Nrxn3βs and membrane-bound Nrxn3β-CTFs. The latter are subsequently cleaved in the transmembrane domain by γ-secretase to generate multiple P/Aβ-like fragments and a single ICD. Knowing that ADAM10 also processes other substrates implicated in neuronal functions (including APP^16^, NL1^11^ or Notch^17^) and given the fact that the Nrxn3β isoform is abundantly expressed throughout the brain, including the hippocampus^18^, we decided to explore if the production of sNrxn3β by ADAM10 affected neuronal development and spine formation in mice hippocampal newborn neurons (NBNs) *in vivo*. Indeed, we observed that sNrxn3β secreted by mature neurons increased spine density on NBNs, whereas the expression of sNrxn3β by NBNs affected their own axonal development.

## Results

### Determination of the sheddase cleavage sites in Nrxn3β

**Figure 1:**
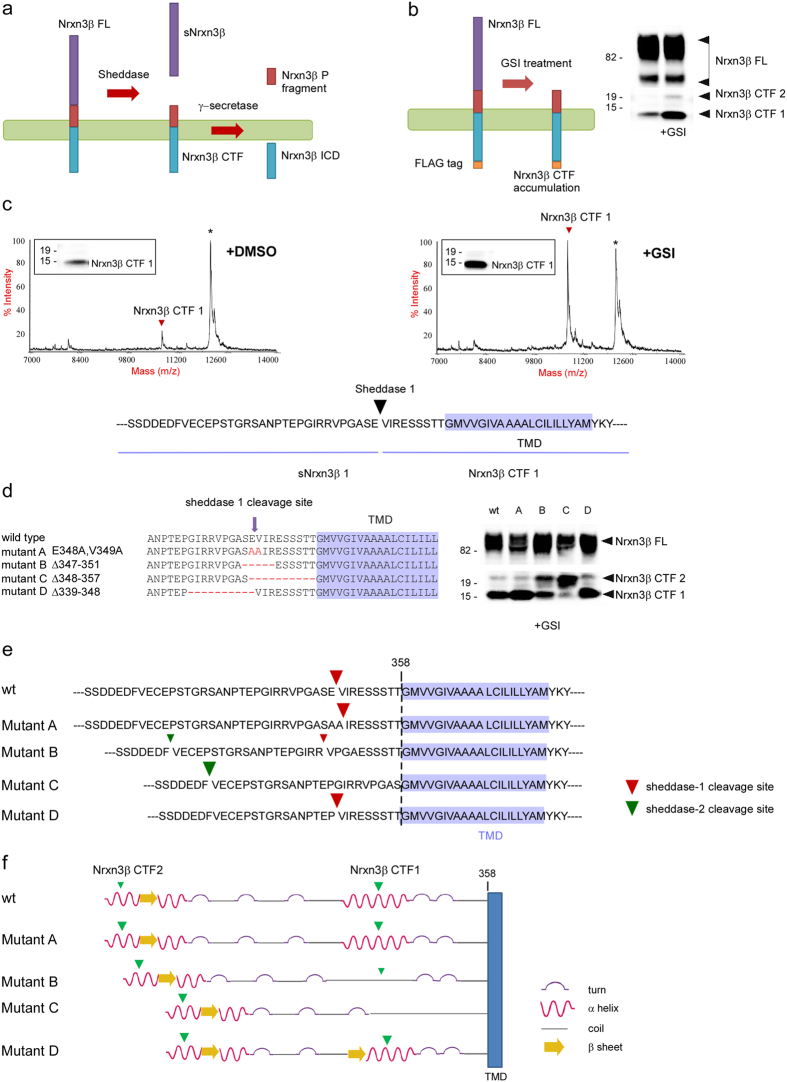
Characterization of the neurexin 3β shedding sites. (**a**) Shedding of Nrxn generates a C-terminal fragment (CTF), which is further processed by γ-secretase resulting in the production of intracellular domain (ICD) and P fragments. (**b**) Treatment of cells expressing Nrxn3β-FLAG with the γ-secretase inhibitor (GSI) Compound E results in the accumulation of CTFs. Total protein extracts were immunostained with an anti-FLAG antibody. (**c**) Mass spectrometric determination of Nrxn3β CTF1 sequence. Nrxn3β-FLAG CTF1 was immunoprecipitated using the M2 resin and analyzed by MALDI-TOF MS. (**d**) Mutants were generated in order to abolish neurexin 3β cleavage at the sheddase 1 site. Mutants A and D cleavage resulted in the generation of mainly CTF1, whereas mutants B and C are characterized by increased generation of CTF2. (**e**) Sheddases cleavage sites of the mutants. Size of the arrow indicates the relative amounts of the CTFs generated. TMD: transmembrane domain. (**f**) Summary diagram showing the secondary structure predictions of Nrxn3β wt and mutants and the position of the sheddase cleavage sites. Note that sequence modifications in mutants B and C favor the rearrangement of the sequence from an α-helix to a coil configuration, harboring the CTF1 sheddase site. Size of the arrow indicates the relative amounts of CTFs generated. The full-length blots are shown in Supplementary Fig. S1.

Cleavage of full length (FL) Nrxn3β by sheddases generates C-terminal fragments (CTFs) that are further cleaved by γ-secretase, resulting in the generation of an intracellular domain (ICD) and extracellular fragments (Fig. 1a). To determine the sites at which Nrxn3β is processed by sheddases, cells expressing Nrxn3β were incubated with a γ-secretase inhibitor (GSI), which resulted in the intracellular accumulation of a major 16 kDa Nrxn3β-CTF fragment (Nrxn3β-CTF1) and a minor 19 kDa fragment (Nrxn3β-CTF2) (Fig. 1b). Next, the primary sequence of the Nrxn3β-CTF1 was determined by immunoprecipitation combined with mass spectrometry (IP/MS) (Fig. 1c), revealing a sheddase-1 cleavage site at residues E348-V349 in the human Nrxn3β sequence (Fig. 1c).

To produce cellular amounts of Nrxn3β-CTF2 compatible with IP/MS identification of its sequence, we forced the cleavage at the sheddase-2 site by generating Nrxn3β mutants in which the sheddase-1 cleavage site E348-V349 was either mutated or deleted (mutants A–D, Fig. 1d).

Surprisingly, alanine substitutions or deletion of residues preceding the sheddase-1 cleavage site did not affect the processing of Nrxn3β at this site (Fig. 1d). In contrast, the deletion of residues following or including the cleavage sequence (mutants B and C) caused a drastic reduction in CTF1 production and an increased production of CTF2 (Fig. 1d). IP/MS analysis of the latter revealed fragments starting at residues ‘VECEPS’ (Fig. 1e & Supplementary Fig. S2), corresponding to a sheddase-2 cleavage site between residues F322 and V323 of the human Nrxn3β sequence (Fig. 1e). Interestingly, secondary structure prediction analysis showed that CTF1 and CTF2 cleavage sites are located in helical regions of Nrxn3β (Fig. 1f). Therefore, destabilization of the helical region at the sheddase-1 cleavage site (mutants B and C) reduced the CTF1 production. Altogether, our results show that in the human Nrxn3β, sheddase-1 and sheddase-2 cleavage sites are E348-V349 and F322-V323, respectively, and that both the secondary structure of the sheddase-1 cleavage site and its distance from the membrane are important determinants for Nrxn3β shedding.

### The metalloproteases ADAM10 and ADAM17 are shedding Nrxn3β

**Figure 2:**
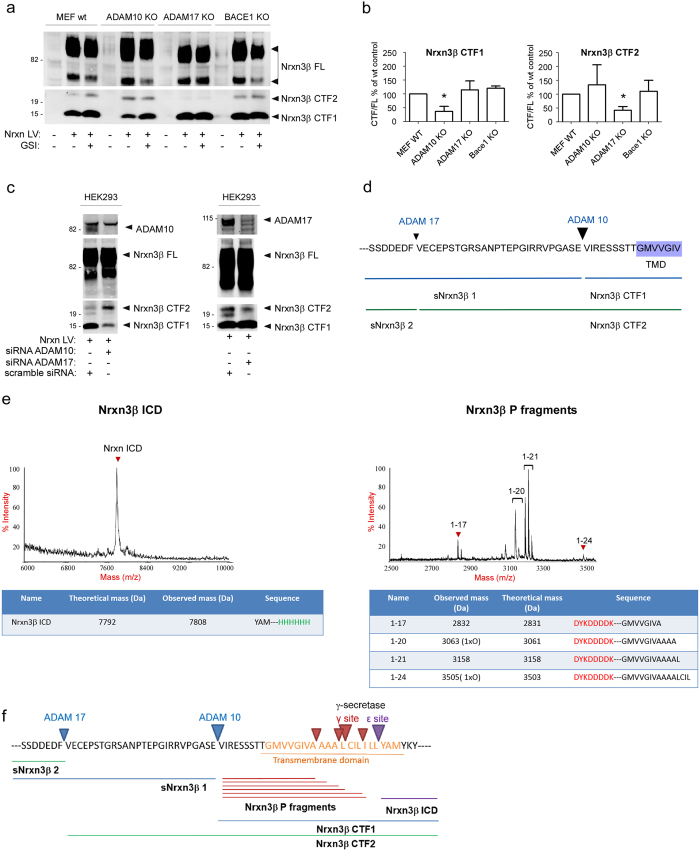
Identification of the enzymes shedding neurexin 3β and characterization of the γ-secretase cleavage sites in its TMD. MEF wt, ADAM10 KO, ADAM17 KO and BACE1 KO cells were infected with Nrxn3β-FLAG-expressing lentivirus and treated with 10 μM of the GSI Compound E or DMSO (control). Cells were collected for CTF1 and CTF2 detection by Western blot (**a**) and densitometric quantification (**b**). CTF levels were normalized to the FL levels. Drastic reductions in the amounts of CTF1 (t(4) = 3.33, P = 0.029) and CTF2 (t(4) = 3.43, P = 0.026) were observed in ADAM 10 KO and ADAM 17 KO MEFs, respectively. (**c**) Nrxn3β CTF1 and CTF2 are generated by ADAM10 and ADAM17, respectively. HEK293 cells transfected with Nrxn3β-FLAG were treated with siRNAs targeting ADAM10, ADAM17 or scramble siRNAs (control). A reduction in the amount of CTF1 was observed in cells treated with ADAM10 siRNAs, whereas a reduction in the amount of CTF2 was found in cells treated with ADAM17 siRNAs. (**d**) Summary diagram showing the ADAM10 and ADAM17 cleavage sites in Nrxn3β. Size of the arrow indicates the relative amounts of CTFs. (**e**) Processing of the double-tagged γ-secretase substrate FLAG-Nrxn3β-CTF1-His7 used in a cell-free assay with purified γ-secretase. MS spectra show the peaks corresponding to Nrxn3β ICD (left) and the Nrxn3β P-fragments (right). (**f**) Summary diagram of the Nrxn3β cleavage sites and the resulting cleavage products. TMD: transmembrane domain. *P < 0.05. Error bars represent s.e.m. The full-length blots are shown in Supplementary Fig. S4.

We next identified the enzymes responsible for Nrxn3β shedding by using multiple genetic methods. First, over-expression of Nrxn3β in mouse embryonic fibroblast (MEFs) cell lines lacking ADAM10^19^, ADAM17 and BACE1^20^ revealed a drastic reduction in the production of Nrxn3β-CTF1 in MEFs lacking ADAM10, and a drastic reduction in the Nrxn3β-CTF2 production in MEFs lacking ADAM17. BACE1 KO MEFs did not exhibit any cleavage phenotype (Fig. 2a,b). Next, we confirmed these data in HEK293 cells incubated with siRNAs targeting ADAM10 and ADAM17 (Fig. 2c). Altogether, our results clearly indicate that the main sheddases responsible for the generation of Nrxn3β-CTF1 and CTF2 are ADAM10 and ADAM17, respectively (Fig. 2d).

### γ-Secretase processing sites in Nrxn3β

We have previously shown that the Nrxn3β-CTFs generated by the sheddases are further processed in their transmembrane region by γ-secretase^8^. The exact sites at which the Nrxn3β main CTF is cleaved by γ-secretase were further determined by using a cell-free assay performed with purified γ-secretase and a double-tagged FLAG-Nrxn3β CTF1-His7 substrate (Supplementary Fig. S3). IP/MS analysis of the cleavage products revealed the generation of a single 57 amino acid long Nrxn3β-ICD (cleavage site: L375-Y376) and multiple P-fragments (cleavage sites: A365-A366, A368-L369, L369-C370, L372-I373) (Fig. 2e). The full metalloprotease and γ-secretase processing scheme is now available for Nrxn3β (Fig. 2f).

### Nrxn3β secreted by hippocampal NBNs decreases the number of axonal extensions without affecting neuronal arborisation, spine density and shape

**Figure 3:**
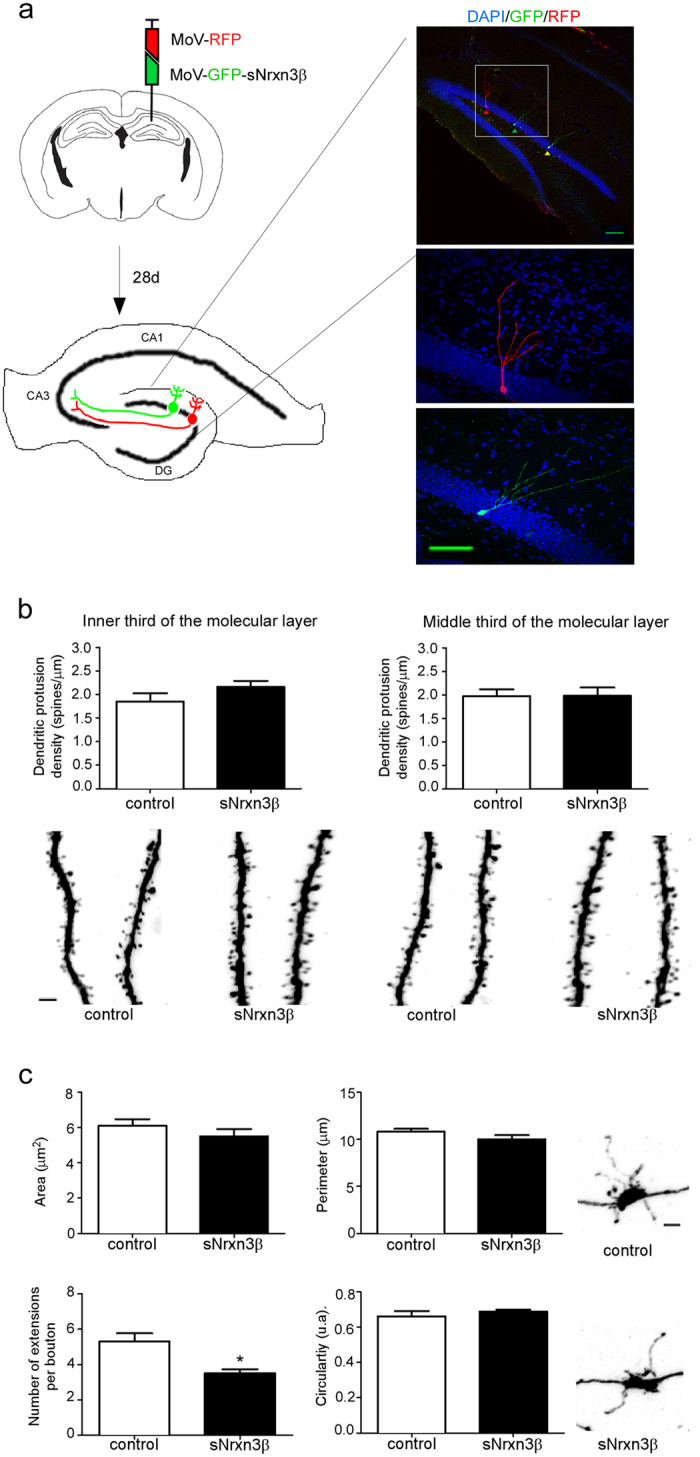
Soluble neurexin 3β secreted by hippocampal newborn neurons affects axonal filopodia development at 28 d.p.i. (**a**) A mix of control-RFP and sNrxn3β-GFP retroviruses was injected into the dentate gyrus of 7-week-old mice: control NBNs in red and sNrxn3β-overexpressing in green and yellow (10X scale bar of 100 mm; 40X, scale bar of 25 mm). (**b**) Summary of spine density quantification from control and sNrxn3β expressing cells (n = 4–5 animals per group, 21–29 dendritic segments per group and third). Representative images of dendritic segments (scale bar 2 μm) are shown. (**c**) Summary graphs of area, perimeter, number of filopodial extensions and circularity (U = 0, P = 0.016) of the MFTs and confocal micrographs of representative axonal boutons for each condition (n = 4–5 animals per group, 11–16 boutons per mice). *P < 0.05 *versus* control group. Error bars represent s.e.m. DG: dentate gyrus, MoV: Moloney virus.

Adult hippocampal newborn neurons (NBNs), with their intense synaptogenesis occurring in discrete populations of cells^21^,^22^, provide a potent model for investigating *in vivo* synaptogenesis and neuronal development. In order to analyze whether the major sNrxn3β secreted by NBNs was able to affect NBNs development, we injected Moloney retroviruses expressing sNrxn3β into the dentate gyrus of 7-week-old mice. These viruses targeted a birth-dated cohort of newly-divided cells in the subgranular zone of the dentate gyrus^21^ and allowed us to study the cell-autonomous effect of sNrxn overexpression. As shown in Fig. 3a, two different Moloney retroviruses were injected, resulting in GFP+ and double-labelled GFP+/RFP+ neurons identified as adult NBNs expressing sNrxn3β, and in RFP+ control NBNs cells. The expression of the GFP-2A-sNrxn3β construct was confirmed *in vitro* (Supplementary Fig. S5a–g). At 28 d.p.i., we found no effect of sNxrn3β on dendritic extension, length of the dendritic tree and complexity of the dendritic arbor by Sholl analysis (Supplementary Fig. S6a). Next, to evaluate changes in synaptic connectivity induced by sNrxn3β on adult NBNs we analysed the density and the shape of the dendritic protrusions both in the inner third (innervated mainly by hilar/commissural axons) and in the middle third (innervated mainly by entorhinal axons) of the molecular layer. No effect of sNrxn3β was found in any of the segments analysed (Fig. 3b and Supplementary Fig. S6b).

Dentate gyrus granule neurons project their axons to CA3, establishing well-defined excitatory connections with pyramidal neurons^23^ called mossy fiber terminals (MFTs). We studied the effect of sNrxn3β on NBNs axonal connections by estimating the number of projections, and importantly, we found that sNrxn3β-overexpressing MFTs presented fewer extensions in comparison with control terminals at 28 d.p.i., although they did not differ in shape and size (Fig. 3c). Together, these findings show that the production and release of sNrxn3β by the presynaptic terminals of NBNs is inhibiting the presynaptic maturation of the axonal protrusions, without affecting spine generation or maturation.

### An environment enriched in sNrxn3β increases dendritic spine density in adult-born neurons *in vivo*

**Figure 4:**
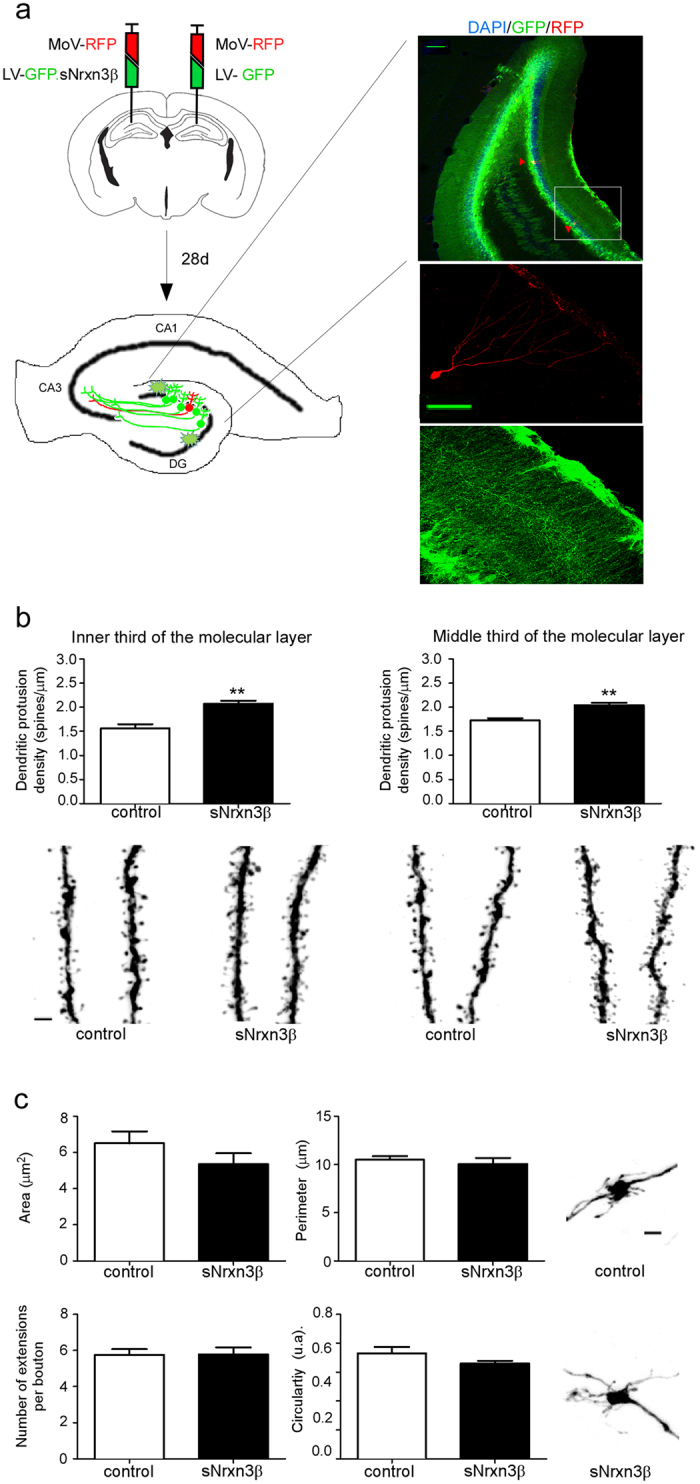
Soluble neurexin 3β released by granule cells exerts a spinogenic effect on newborn neurons at 28 d.p.i. (**a**) Seven-week-old animals were injected in the dentate gyrus of the right hemisphere (control hemisphere) with a mix of control-RFP Moloney viruses and control-GFP lentiviruses, and in the dentate gyrus of the left hemisphere (sNrxn3β expressing hemisphere) with a mix containing control-RFP-Moloney viruses and lentiviruses carrying the sNrxn3β-GFP expression cassette. 28 days after injection, we studied the morphology of RFP+/GFP− newborn neurons growing in the absence (control condition) or in the presence of secreted neurexin-3β. Representative images show NBNs (in red) in the dentate gyrus surrounded by cells expressing sNrxn3β (in green) (10X scale bar of 100 mm; 40X scale bar of 25 mm). (**b**) Quantitative analysis of spine density of the inner (t(7) = 5.104, P = 0.0014) and middle thirds (t(7) = 3.835, P = 0.006)) of the molecular layer (n = 4–5 animals per group, n = 30–39 dendritic segments per group and per third of the molecular layer) and confocal images of dendrites. (**c**) Summary graphs of the area, perimeter, number of filopodial extensions and circularity of the MFT and confocal micrographs of representative MFTs for each condition (n = 4–5 animals per group, 16–27 boutons per mice). Error bars represent s.e.m. **P < 0.01 *versus* control group. Error bars represent s.e.m. DG: dentate gyrus. MoV: Moloney virus, LV: lentivirus.

The finding that sNrxn3β produced by NBNs only affected their presynaptic development encouraged us to study whether a sNrxn3β-enriched environment (created by sNrxn3β-secreting cells surrounding the NBNs) would have the same effect on NBNs morphology. To test this hypothesis, a mix of lentivirus and Moloney virus was injected into the dentate gyrus of the hippocampus (Fig. 4a). At 28 d.p.i., we found no effect of sNrxn3β on neuronal arborisation (Supplementary Fig. S7a) or on the percentage of small, medium and large spines (Supplementary Fig. S7b). However, spine density was significantly increased by sNrxn3β in the inner and in the middle thirds of the molecular layer of NBNs (Fig. 4b). Surprisingly, the inhibitory effect on axonal development of sNrxn3β released by NBN disappeared when NBNs developed under the sNrxn3β-enriched environment (Fig. 4c). Taken together, these results demonstrate that the high levels of sNrxn3β in the dentate gyrus induced an increase in the number of dendritic spines in the NBNs without affecting their spine morphology or axonal development.

## Discussion

In the present study, we determined the sites at which Nrxn3β is processed and identified ADAM10 and ADAM17 as the main enzymes responsible for the generation of the short and long Nrxn3β-CTFs, respectively (Fig. 2b). Based on the observation that some Nrxn3β-CTF1 fragments were apparently still produced in the ADAM10 KO cells (Fig. 2a), it remains possible that other secretases/metalloproteases may be involved in the proteolytic process of Nrxn3β functioning, in a competitive or associative manner. Altogether, our results reveal interesting similarities and differences with APP and Notch processing patterns. In case of APP, the α-secretase processing by ADAM10 or ADAM17 results in the generation of the short CTF (APP-C83), whereas the processing by β-secretase (BACE1) results in the generation of the long CTF (APP-C99)^24^. Similarly to APP, we found that Nrxn3β shedding leads to the formation of two CTFs and that the ADAM10 processing occurs in α-helical regions and depend on the distance from the membrane rather than the precise sequence at the cleavage site (Fig. 1e–f). The shedding of the Notch1 receptor is particular because its cleavage by ADAM10 requires the binding of a ligand, whereas ADAM17 is involved in a ligand-independent processing^17^. In contrast to Nrxn3β, the shedding of Notch by the two enzymes occurs at the same site, leading to the formation of one single CTF^17^. Characterization of the γ-secretase processing sites of Nrxn3β revealed the presence of a unique ICD fragment and multiple P3-like fragments (Fig. 2e), similar to Notch processing that generates one ICD, but different from APP processing that results in two ICDs^25^. Another similarity to other γ-secretase substrates like APP, Notch1 and CD44, is that the Nrxn3β site generating ICD (ε site) was located close to the cytosolic end of the transmembrane region, three residues into the membrane^26^. Very interestingly, we found a significant conservation of particular residues at the ADAM17, ADAM10 and the γ-secretase cleavage sites in the neurexin isoforms 1α, 1β, 2α, 2β, 3α and 3β (Supplementary Fig. S8) suggesting that most Nrxn isoforms are processed in a similar manner.

**Figure 5:**
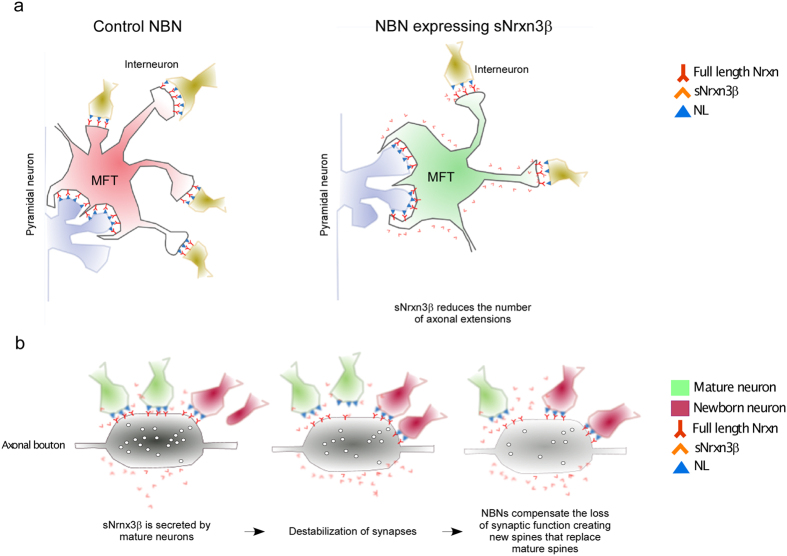
Models for sNrxn3β functions in spinogenesis and axonal development. (**a**) Possible effect of sNrxn3β on axonal protrusions. Left panel represents the axonal terminal of a control NBN. Right panel shows how soluble neurexin released by NBNs’ mossy fiber terminals induces a decrease in the number of axonal protrusions without affecting the shape or the size of the boutons. (**b**) sNrxn3β released by granule cells may destabilize the Nrxn/NL complexes affecting the presynaptic function. This alteration would lead to the increase in spine number of NBNs in order to compensate a general altered function in the hippocampus, replacing the spines of the mature neurons.

Given the variety of Nrxn functions at the synapse^1^, we next investigated whether the cleavage fragments resulting from Nrxn processing by ADAM10 could play a role in synapse function and development *in vivo*. Indeed, the physiological contribution of soluble neurexin’s to animal neural network functioning is poorly understood, mainly because their roles in synapse formation have been studied *in vitro* on culture models^27^,^28^,^29^. *In vitro,* sNrxn can act as a competitor of endogenous full-length neurexin disrupting the Nrxn/NL complexes, thus destabilizing newly formed synaptic contacts and affecting presynaptic function^28^,^30^,^31^,^32^,^33^. To get new insights into the physiological functions of sNrxn3β, we studied its effect *in vivo* on neuronal development and spinogenesis. In our experimental model, we observed a negative cell-autonomous effect of sNrxn3β on the number of NBN axonal filopodia (Fig. 3c) that it is known to establish contacts with inhibitory interneurons^34^. It is therefore possible that secretion of sNrxn3β from the NBNs’ axonal terminals interferes with endogenous Nrxn function, altering the stabilization of axonal extensions through a yet uncharacterized mechanism that could involve neuroligin and that might destabilize synaptic interactions (Fig. 5a). The reason why the large boutons’ size or morphology have not been affected by the expression of sNrxn could be related to the different structural and functional properties described between axonal extensions and boutons^34^,^35^. In fact, in epilepsy models, the number of filopodial extensions is reduced, while the connection to pyramidal cells remains intact^35^.

In sharp contrast, NBNs that grew in the environment of neighboring cells secreting sNrxn3β exhibited increased spine density (Fig. 4b), without any effect on mossy fiber terminals (Fig. 4c). During development, NBNs integrate into the pre-existing neuronal network and their dendritic spines compete with those of mature neurons, replacing them eventually^36^. Knowing that a reduction in synaptic activity has been observed in response to sNrxn1β addition to primary neuronal cultures^28^,^31^, sNrxn3β released by mature neurons may alter the general function of the hippocampus by affecting the synaptic strength and the presynaptic function of hippocampal neurons. Therefore, the increased spine density found in NBNs growing in a sNrxn3β-enriched environment could reflect an attempt of these newly generated cells to repair or compensate a synaptic dysfunction based on their competitive advantage in synapse formation (Fig. 5b). In fact, it has also been shown that NBNs respond to the lesion of the perforant path by creating more spines^37^.

Together, our results establish a major step towards a better understanding of the neurobiological roles of the proteolytic processing of synaptic cell adhesion molecules, suggesting a pivotal role of sNrxn in plasticity and network remodelling during neuronal development.

As a general picture, we and others have provided evidence that supports the connection between neuronal activity, the proteolytic processing by different secretases of the synaptic cell adhesion molecules neurexins, and synaptic plasticity or neuronal development. First, stimulation *in vitro* of neuronal activity favors the processing of neurexin 3β by an α-secretase and therefore increases the release of the soluble extracellular domain^8^. It has also been shown that the soluble neurexin-1β binds post-synaptic neuroligin in primary cortical neurons triggering its own cleavage and release of soluble neuroligin-1^11^ that might destabilize synaptic interactions. We show here that sNrxn3β plays a function in plasticity and network remodeling during neuronal development. Finally, the last step in the successive proteolytic processing of neurexin3β by α- and γ-secretases releases an intracellular domain (neurexin 3β-ICD) that may translocate into the nucleus and regulate the expression of genes having important neurobiological functions that includes synaptogenesis.

It is thus tempting to hypothesise that under pathological conditions such as schizophrenia, autism spectrum disorders or Alzheimer’s disease, alterations of Nrxn processing may contribute to weakening of particular connections leading to imbalanced neuronal networks. The concept of targeting Nrxn processing for therapeutic applications to treat these brain disorders needs to be evaluated.

## Methods

### Animals

Animals used were 7-week-old C57BL6/J male mice. Mice were group-housed in standard cages under light- (12 h light/dark cycle) and temperature-controlled (22 °C) conditions. The maximal number of mice per cage was 5. Food and water were available *ad libitum*. Every effort was made to minimize the number of animals used and their suffering. All *in vivo* procedures were carried out in accordance with institutional guidelines of Canton Vaud. Experimental protocols were approved by the Swiss animal experimentation authorities (Service de la consommation et des affaires vétérinaires, Epalinges, Switzerland, Authorization numbers: 2302, 1986 and 1980.1).

### Expression vectors

For the *in vitro* studies, genes of interest have been subcloned into the pCDNA 3/Neo (+) (Invitrogen) vector for expression in mammalian cell lines. Human Nrxn3β-FLAG generation has been described before^8^. The Nrxn3β sequence used can be found in Uniprot database (reference number Q9HDB5-2). Nrxn3β-FLAG, ADAM10 and ADAM17 constructs have been subcloned into the pSin lentiviral transfer vector for generation of lentiviruses. For bacterial expression and protein purification, double-tagged (FLAG tag at the N-terminus and His tag at the C-terminus) Nrxn3β CTF has been subcloned into the pet21b vector (Novagen).

For infecting specifically newborn neurons *in vivo* we used retroviral constructs containing a red or green fluorescent protein (RFP or GFP respectively)-expression cassette under the control of the CMV early enhancer and chicken beta-actin promoter as previously described^21^,^38^. The construct encoding the soluble part of Nrxn3β (cag-GFP-2A-sNrxn3β) was subcloned into the mentioned backbone vector. The same construct was subcloned into the pBob lentiviral vector under the CMV promoter. A pBob-GFP lentiviral vector was used as control.

### Deletion mutant generation

Neurexin deletion mutants were generated as previously described^39^. Briefly, phosphorothioate residues were incorporated into the regions flanking the desired deletion sequence using PCR amplification reaction in a Biometra Thermocycler. Pfu Turbo DNA polymerase (Agilent) was used for the reaction to assure higher proofreading ability. Following the amplification, the product was treated with DpnI endonuclease (Roche, Basel, Switzerland). The blunt ends were digested using T7 exonuclease (Bioconcept, Allschwil, Switzerland), which led to the generation of 3′ complementary overhangs at both ends of the PCR product. Subsequently the PCR product underwent self-ligation. DH10B cells were transformed with the obtained DNA.

### Transfections

Genes of interest were introduced into the cells using the calcium phosphate transfection method. Cells were then incubated at 37 °C, 5% CO2 and after 6 h, fresh medium (DMEM with 10% fetal bovine serum and 1% penicillin/streptomycin) was added to cells. After 48 h, whole cell extracts were generated and proteins were separated by electrophoresis on the NuPAGE^®^Novex^®^ 4–12% Bis-Tris gels (Invitrogen) for SDS-PAGE analysis.

### siRNA knockdown

ADAM10 and ADAM17 were knocked down using the ON-TARGET*plus* SMARTpool siRNA (Dharmacon). Allstars negative siRNA control (Qiagen, AG, Base, Switzerland) was used as a control. siRNAs were delivered to the cells using the Lipofectamine RNAiMAX reagent (Invitrogen) following the manufacturer’s instructions.

### Virus production

Lentiviral production has previously been described^40^. pSin vectors were co-transfected with plasmids encoding viral particles (pMD2G and pCMVR8.74) whereas pBob vectors were co-transfected with third generation helper plasmids (pRSV-REV, pMDLg/pRE and VSVG). Titrations were performed by qPCR (7900HT thermocycler, Applied Biosystems) as previously described^40^. Production and titration of the Moloney viruses was performed as previously described^41^. Final titers were between 10^8^–10^10^ vg/mL depending on the lentivirus and between 10^7^–10^9^ pfu/mL depending on the Moloney virus.

### Total protein cell extracts

Cells were first washed with PBS (1x, Gibco) and lysed with HEPES NP-40 lysis buffer (50 mM HEPES, 150 mM NaCl, 5 mM MgCl_2_, 5 mM CaCl_2,_ 1% Nonidet P-40, 1x protease inhibitor cocktail (Complete, Roche)) and then scraped and incubated on ice for 1 h. Samples were centrifuged for 1 h at 13,000xg, at 4 °C. Protein concentrations were normalized using the Pierce^TM^ BCA Protein Assay Kit (Thermo Scientific).

### Protein expression and purification

For expression of double-tagged (FLAG-His) neurexin substrate, E. *coli* strain BL21 (Invitrogen) was transformed using the gene of interest subcloned into the pet21b expression vector (Novagen). The expression of proteins was induced for 4 h at 37 °C in 1 litre of LB/ampicillin containing 1 mM isopropyl 1-thio-β-D-galactopyranoside. Cells were lysed with 10 mM Tris pH 7.0, 150 mM NaCl, 1%Triton X-100, and complete protease inhibitor cocktail (Roche) and passed three times through a high-pressure homogenizer (Emulsiflex-C5; Avestin, Inc., Mannheim, Germany) at a pressure greater than 1000 psi. The proteins recovered were incubated overnight with the M2 anti-FLAG affinity resin (Invitrogen) and the bound proteins were eluted in batch using the acidic solution (1%NP40, 100 mM glycine at pH2.7). The elution fractions were pulled together and bound overnight to the Nickel beads (Invitrogen). The proteins were then eluted using 200 uM imidazole solution. The purity of the eluted material was confirmed by SDS-PAGE using Coomassie blue staining (Applichem, MO, USA).

### γ- Secretase activity assay

The *in vitro* activity assay was performed using the purified double-tagged substrate and purified γ-secretase. Purified γ-secretase was solubilized in 0.2% (wt/vol) CHAPSO, 50 mM HEPES (pH 7.0), 150 mM NaCl, 5 mM MgCl_2_ and 5 mM CaCl_2_, and incubated at 37 °C for 4 h with 1 μM substrate, 0.1% phosphatidylcholine (PC), 0.025% phosphatidylethanolamine (PE). Compound E (Milipore) was added in control reactions. The samples were then run on NuPAGE^®^ Novex^®^ 4–12% Bis-Tris gels (Invitrogen) for SDS-PAGE analysis and transferred onto the PVDF membranes (Bio-Rad). Membranes were boiled and probed using anti-His and anti-FLAG (M2) antibodies in order to respectively detect the C- and N- termini of the processed substrate.

### Reverse transcription-polymerase chain reaction (RT-PCR) and Northern blotting

Total RNA from transfected HEKs and DIV14 infected neurons was extracted using an RNeasy kit (Qiagen, Hilden, Germany). The first-strand cDNA was synthesized from 1 μg or 100 ng of total RNA of HEKs and neurons respectively using the ImProm-II™ Reverse Transcription System Kit (Promega, Madison WI, USA). Specific primers that recognise the extracellular part of Nrxn3β (Fw –CGCTCTACAGCCAGCATTC, Rev – GTGGGGTTTGCTGACCTACCT) were used to amplify the obtained cDNA. PCR products were loaded in a 1% agarose gel and stained with Ethidium bromide. RT-PCR was performed using a Biometra Thermocycler.

### Western blotting

20–25 μg of proteins were separated using NuPAGE 4–12% Bis-Tris gel (Novex), transferred onto the nitrocellulose membrane (Whatman) and probed with the corresponding primary and secondary antibodies listed in the Supplementary Table 1. Fluorescent signal was detected using the Odyssey infrared imaging system (LICOR). Densitometric analysis was performed using ImageJ software (imagej.nih.gov/ij).

### TCA protein precipitation

For precipitating proteins, DMEM culture medium was replaced for 24 hours by medium without serum. Before harvesting the cells, 1 ml of culture medium was collected and centrifuged at 1000xg for 3 min. Supernatant was collected and mixed in a 1:1 proportion with 20% trichloroacetic acid (TCA, Sigma, Sigma St. Louis, MO, US) in water. After 30 min on ice, samples were centrifuged for 20 min at 13,000x g, at 4 °C. Supernatants were removed and the pellets were resuspended in cold pure acetone. After a second centrifugation, acetone was removed. Once the pellets were completely dry, they were resuspended in alkaline sample buffer (50 mM Tris pH 8.0, 2% SDS, 100 mM DTT, 10% glycerol) and kept overnight at 4 °C.

### Dot blot analysis

4, 6 and 8 μl of each sample were carefully pipetted onto a nitrocellulose membrane. The membrane was left to dry for 15 min. Non-specific sites were blocked overnight at 4 °C (blocking buffer, Rockland). The presence of soluble Nrx3β was detected by incubating the membrane with the corresponding primary and secondary antibodies (see Supplementary Table 1). ImageJ software (imagej.nih.gov/ij) was used for the densitometric analysis.

### Mass spectrometry

The processed double-tagged substrate was immunoprecipitated overnight at 4 °C using successively M2 anti-FLAG beads and anti-His antibody coupled to Protein G sepharose for the purification of polyHistidine containing proteins (Invitrogen). Neurexin P fragments and ICD were eluted with 1:20:20 (v:v:v) 1% (vol/vol) trifluoroacetic acid:acetonitrile:H_2_O and analysed by MALDI-TOF mass spectrometry in reflectron or linear mode on an ABI 4800 MALDI-TOF/TOF mass spectrometer (Applied Biosystems, Carlsbad, California, USA). Molecular masses were accurately measured and searched against amino acid sequences of human Nrxn3β with addition of a FLAG sequence and methionine at the N-terminus and addition of the His-tag sequence at the C-terminus in case of the FLAG-Nrxn3β CTF1- His recombinant substrate.

### Secondary structure predictions

Secondary structure predictions were performed using the Chou and Fasman secondary structure prediction software (http://www.biogem.org/tool/chou-fasman/).

### Stereotactic injection

For each experiment, 2 ul of a specific combination of two viruses was injected into the dentate gyrus at the following coordinates from the Bregma: anteroposterior −2 mm, lateral 1.75 mm and dorsoventral −2.25 mm. After every injection and throughout the experiment, animals were regularly monitored for their physical recovery in agreement with local and federal directives for animal experimentation. Mice were sacrificed 28 days post-injection.

### Perfusion

4 weeks post–viral injected mice were perfused transcardially with 0.9% saline (wt/vol), followed by 4% paraformaldehyde (PFA, wt/vol). Then, brain samples were postfixed with 4% paraformaldehyde for additional 24 hours. 60 μm coronal sections were prepared using a cryostat.

### Immunohistochemistry and confocal microscopy

The protocol used was previously described^42^. Briefly, we used one-in-two series of brain sections that were stained to amplify the fluorescent protein signals (for antibodies references, see Supplementary Table 1). DAPI (4,6 Diamidino-2-phenylindol) was used to stain nuclei (diluted 1:5000 in 0.1 M PBS). DAPI fluorescence was acquired in the 415–450 nm spectral range and excited at 405 nm, GFP-Alexa 488 was recorded between 515–540 nm and excited at 488 nm, and RFP-Alexa 594 was acquired between 600–630 nm and excited at 594 nm.

Neuronal analysis was performed as previously described^41^. For analysing the maximal dendritic extension, neurons were imaged with a 40x oil lens and a z-step of 2 lm. In order to analyse the morphology of the mossy fiber terminals (MFT) and synaptic spines, axonal buttons and dendrites were imaged with a 63x oil lens and a z-step of 0.38 lm. The area and perimeter of the MFT were measured on maximal intensity projection by tracing their excluding the satellites and filopodia; circularity was calculated with the following formula: circularity = 4 × π × area/(perimeter)^2^. Dendritic spine analysis included spine density (number spines/μm) and the morphological classification of the dendritic spines according to their head width: spines were defined as ‘small’ (less than 0.2 μm), ‘medium’ (between 0.21 μm and 0.55 μm) and ‘big’ (more than 0.55 μm). All analyses were performed using Fiji software (available at http://fiji.sc/). Sholl analyses of dendritic branching were performed using Fiji software with a BIOPBar Sholl/Extender plug-in (Bioimaging and Optics Platform, EPFL, Lausanne). We defined the radius as 10% of the maximal neuronal length to avoid a masking effect due to differences in the size of the cells analysed. All the images were acquired using a Zeiss LSM 700 Invert confocal microscope (Carl Zeiss, Feldbach, Switzerland).

### Primary cultures, viral infection and immunohistochemistry

Preparation of mouse primary cortical neurons (PCNs) was previously described^43^. Neurons were infected at DIV4 and at DIV14 supernatant was collected for checking the expression of sNrxn3β by dot blot. A cover slip placed in the well was removed before collecting the samples for confirming the expression of the vector by the GFP immunofluorescence. Coverslips were washed with PBS, fixed with 4% PFA and incubated with primary antibodies against GFP and MAP-2. Neurons were then washed and incubated with secondary antibodies (see Supplementary Table 1). Images were analysed using a Zeiss Axioplan 2 microscope with a 10x lens.

### Statistical analysis

All values are represented by the average of independent experiments. Densitormetric values were tested for normality using Shapiro-Wilkinson test. When distribution was normal, an unpaired Student t-test was used. Otherwise, nonparametric Mann-Whitney test was applied. Morphology parameters were analyzed using unpaired t-tests with n equal to the number of animals per group. For analyzing the morphology of the spines one way-ANOVA was used followed by a Bonferroni test or T3-Dunnet test when homocesdasicity failed. In all cases, hypothesis testing was two-tailed. All analyses were performed using GraphPad Prism Software version 5.1. (GraphPad Software, San Diego California USA).

## Additional Information

**How to cite this article**: Borcel, E. *et al*. Shedding of neurexin 3β ectodomain by ADAM10 releases a soluble fragment that affects the development of newborn neurons. *Sci. Rep.* **6**, 39310; doi: 10.1038/srep39310 (2016).

**Publisher's note:** Springer Nature remains neutral with regard to jurisdictional claims in published maps and institutional affiliations.

## Supplementary Material

Supplementary Information
