# Supplementary material for: Shedding of neurexin 3β ectodomain by ADAM10 releases a soluble fragment that affects the development of newborn neurons
Source: Sci Rep. 2016 Dec 19;6:39310. doi: 10.1038/srep39310 (PMC5171655; doi:10.1038/srep39310)
Supplement: Supplementary Information [file srep39310-s1.pdf]

Shedding of neurexin 3β ectodomain by ADAM10 releases a soluble fragment that affects the development of newborn neurons

Erika Borcel*[a], Magda Palczynska*[a], Marine Krzisch [b], Mitko Dimitrov[a], Giorgio Ulrich[a], Nicolas Toni [b], Patrick C. Fraering [a, c, 1].

[a] Brain Mind Institute and School of Life Sciences, Ecole Polytechnique Fédérale de Lausanne (EPFL), CH1015 Lausanne, Switzerland.

[b] Department of Fundamental Neurosciences, University of Lausanne (UNIL), CH1015 Lausanne, Switzerland.

[c] Foundation Eclosion, CH1228 Plan-Les-Ouates & Campus Biotech Innovation Park, CH1202 Geneva, Switzerland.

[1]Correspondance should be addressed to Patrick C.

Email: fraeringpatrick@hotmail.com, tel.: 0041786406462

* Both authors contributed equally to this work

Supplementary Figure S1. Full-length blots of Fig. 1

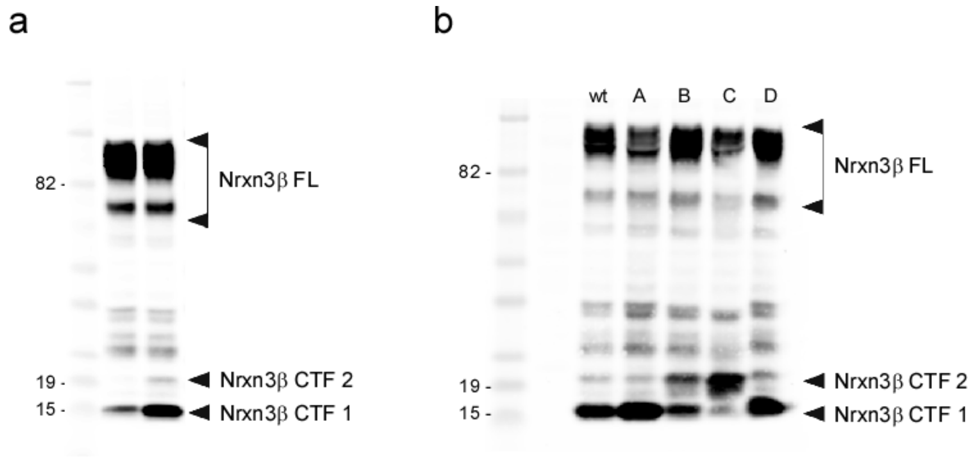

Supplementary Figure S1. Full-length blots of Fig. 1. (a) Full-length blot of Fig 1b. Treatment of cells expressing Nrxn3β-FLAG with the γ-secretase inhibitor (GSI) Compound E results in the accumulation of CTFs. Total protein extracts were immunostained with an anti-FLAG antibody. **(b) Full-length blot of Fig 1d.** Mutants were generated in order to abolish neurexin 3β cleavage at the sheddase 1 site. Total protein extracts were immunostained with an anti-FLAG antibody.

Supplementary Figure S2. Nrxn3β sheddase cleavage sites.

Mutant A

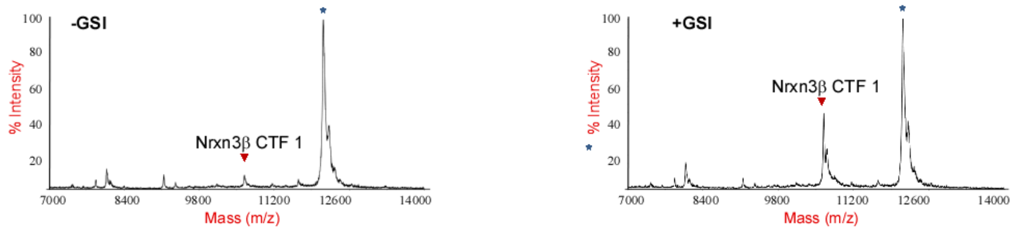

Mutant	Name	Theoretical mass (DA)	Observed mass (Da)	Sequence
A	Nrxn CTF1	10653	10648	IRESSSTTGMVVGIVAAAAL–DYKDDDDK

Mutant B

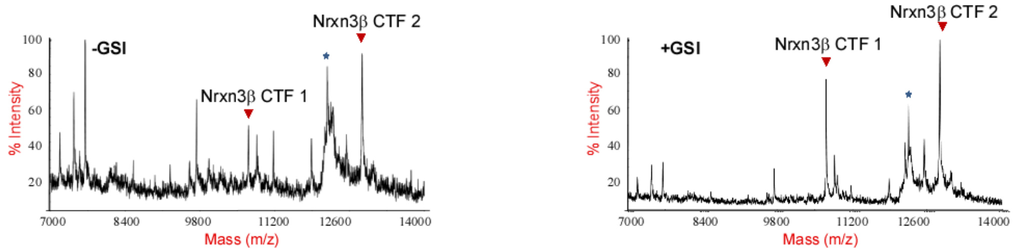

Mutant	Name	Theoretical mass (DA)	Observed mass (Da)	Sequence
B	Nrxn CTF1	12847	12835	VPGAESSSTTGMVVGIVAAAAL-DYKDDDDK
B	Nrxn CTF2	10708	10709	VECEPSTGRSANPTEPGIRRVPGAESSSTTGMVVGIVAAAAL-DYKDDDDK

Mutant C

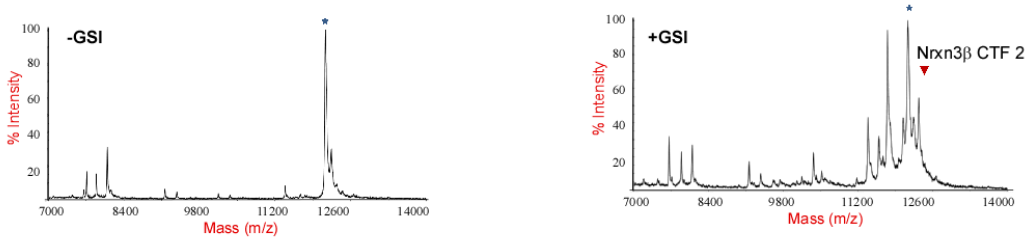

Mutant	Name	Theoretical mass (DA)	Observed mass (Da)	Sequence
C	Nrxn CTF2	12340	12340	VECEPSTGRSANPTEPGIRRVPGAESSSTTGMVVGIVAAAAL-DYKDDDDK

Mutant D

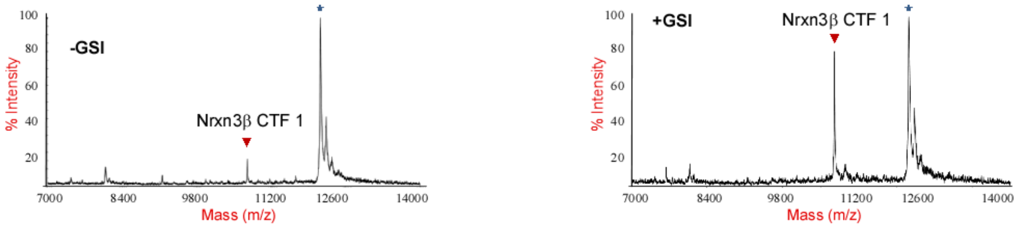

Mutant	Name	Theoretical mass (Da)	Observed mass (Da)	Sequence
D	Nrxn CTF1	10753	10753	VIRESSSTTGMVVGIVAAAALCILILLYAM––DYKDDDDK

Supplementary Figure S2. Nrxn3β sheddase cleavage sites. MS spectra showing the Nrxn3β CTF fragments identified following IP of Nrxn3β wt and mutant CTFs, using the M2 anti-FLAG resin. Masses and sequences identified are shown in the tables. Asterisks indicate non-specific peaks.

Supplementary Figure S3. Purification protocol

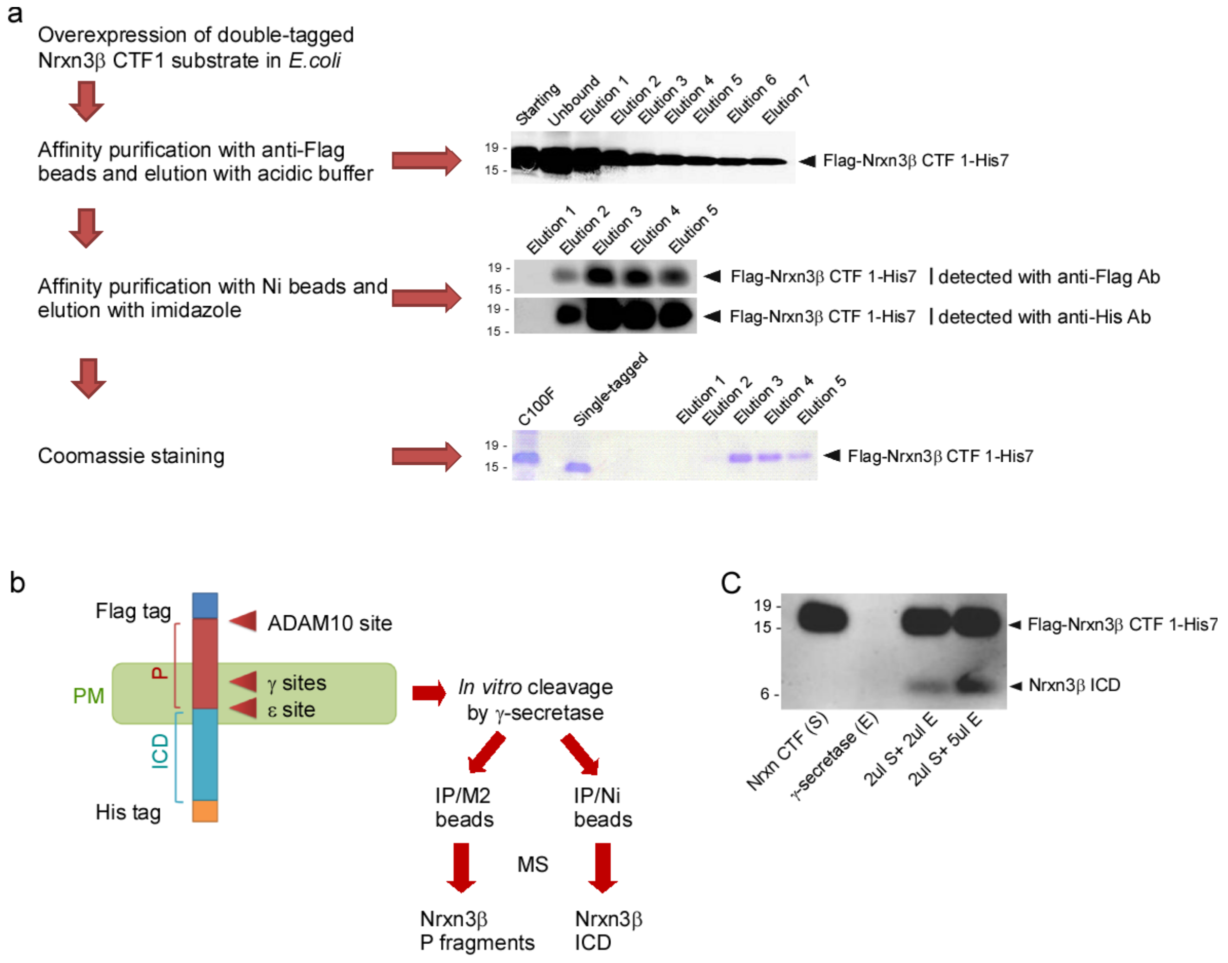

Supplementary Figure S3. Purification protocol (a) Protocol for the purification of the double-tagged FLAG-Nrxn3β-CTF1-His7 substrate. **(b)** Protocol for the analysis of the Nrxn3β γ-secretase cleavage products. **(c)** Dose-dependent generation of the Nrxn3β-ICD fragment as revealed by western blot after cleavage by purified γ-secretase (E) of the FLAG-Nrxn3β-CTF1-His7 substrate (S).

Supplementary Figure S4. Full-length blots of Fig. 2.

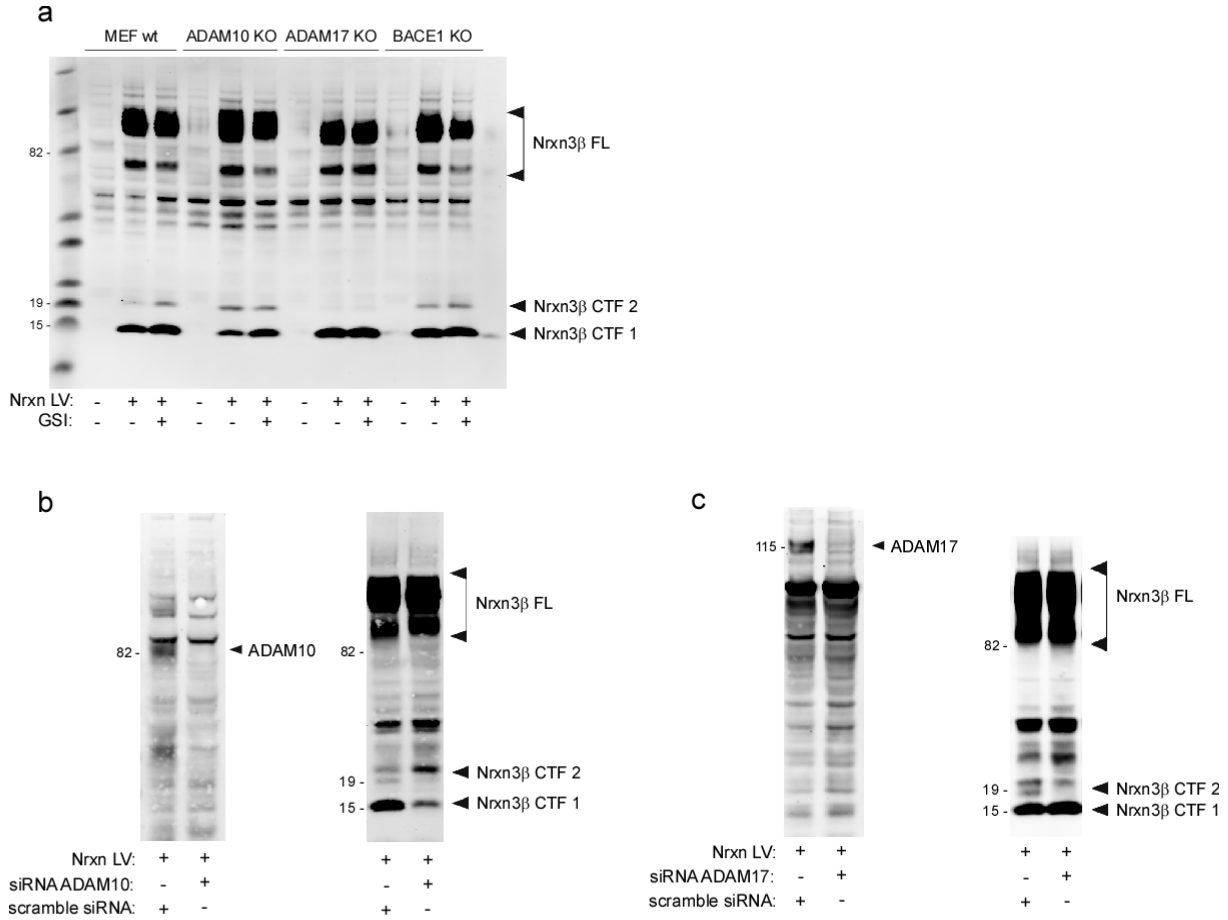

Supplementary Figure S4. Full-length blots of Fig. 2. (a) Full-length blot of Fig 2a. MEF wt, ADAM10 KO, ADAM17 KO and BACE1 KO cells were infected with Nrxn3β-FLAG-expressing lentivirus and treated with 10 µM of the GSI Compound E or DMSO (control). Cells were collected for CTF1 and CTF2 detection by Western blot. **(b) Full-length blot of Fig 2c (left panel).** Nrxn3β CTF1 is generated by ADAM10, respectively. HEK293 cells transfected with Nrxn3β-FLAG were treated with siRNAs targeting ADAM10 or scramble siRNAs (control). **(c) Full-length blot of Fig 2c (right panel).** Nrxn3β CTF2 is generated by ADAM10, respectively. HEK293 cells transfected with Nrxn3β-FLAG were treated with siRNAs targeting ADAM17 or scramble siRNAs (control).

Supplementary Figure S5. Detection of sNrxn3β in HEK cells and neurons.

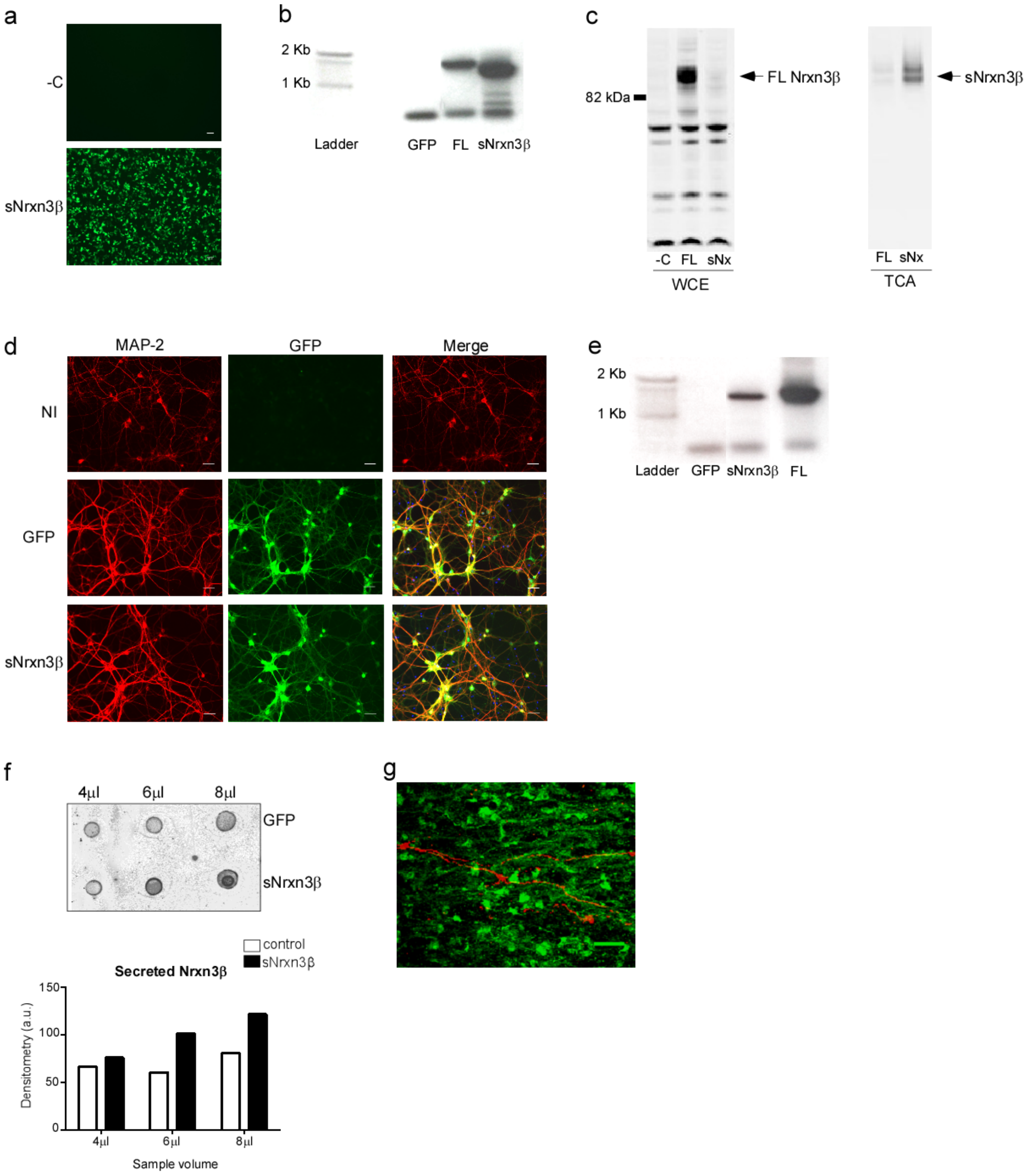

Supplementary Figure S5. Detection of sNrxn3β in HEK cells and neurons. (a) Expression of the vector containing the GFP-2A-sNrx3β vector in transfected HEK cells (20X scale bar of 50 μm). **(b)** RT-PCR products from RNAs extracted from HEK cells transfected

with GFP-2A-sNrxn3β, GFP-2A-full-length (FL) Nrxn3β (positive control) or GFP empty vectors (negative control). **(c)** Western blots confirming the expression and release of sNrxn3β in the culture medium after transfection of HEK cells with a GFP-2A-sNrxn3β plasmid. *Left panel:* whole cell extracts (WCE) of non-transfected cells (-C; Negative control), cells expressing full-length Nrxn3β (FL; Positive control) or cells expressing GFP-2A-sNrx3β (sNX). *Right panel:* Secreted sNrxn3β detected in culture media of cells expressing full-length Nrxn3β (FL) or GFP-2A-sNrx3β (sNX), after protein precipitation by Trichloroacetic acid (TCA). **(d)** Primary cortical neurons non-infected (NI) or infected with either a control-GFP lentivirus (GFP) or the sNrx3β expressing lentivirus (sNrxn) (20X scale bar of 50 μm). **(e)** RT-PCR amplification of sNrxn in RNAs from primary neurons infected with lentiviruses encoding for GFP-2A-sNrxn3β or GFP-2A-FL-Nrxn3β (positive control) or a vector containing only GFP (negative control). **(f)** Dot blot analysis from neurons overexpressing either sNrxn3β or the control GFP (top panel). Bottom panel shows the densitometric analysis. sNx: sNrxn3β. **(g)** Confocal picture showing a NBN axonal terminal (in red) surrounded by mature granule neurons' MFTs (green). 40X; scale bar 10 μm. Negative control (-C): non-transfected cells.

Supplementary Figure S6. **Sholl and spine morphology analysis of NBNs expressing sNrxn3β at 28 d.p.i.**

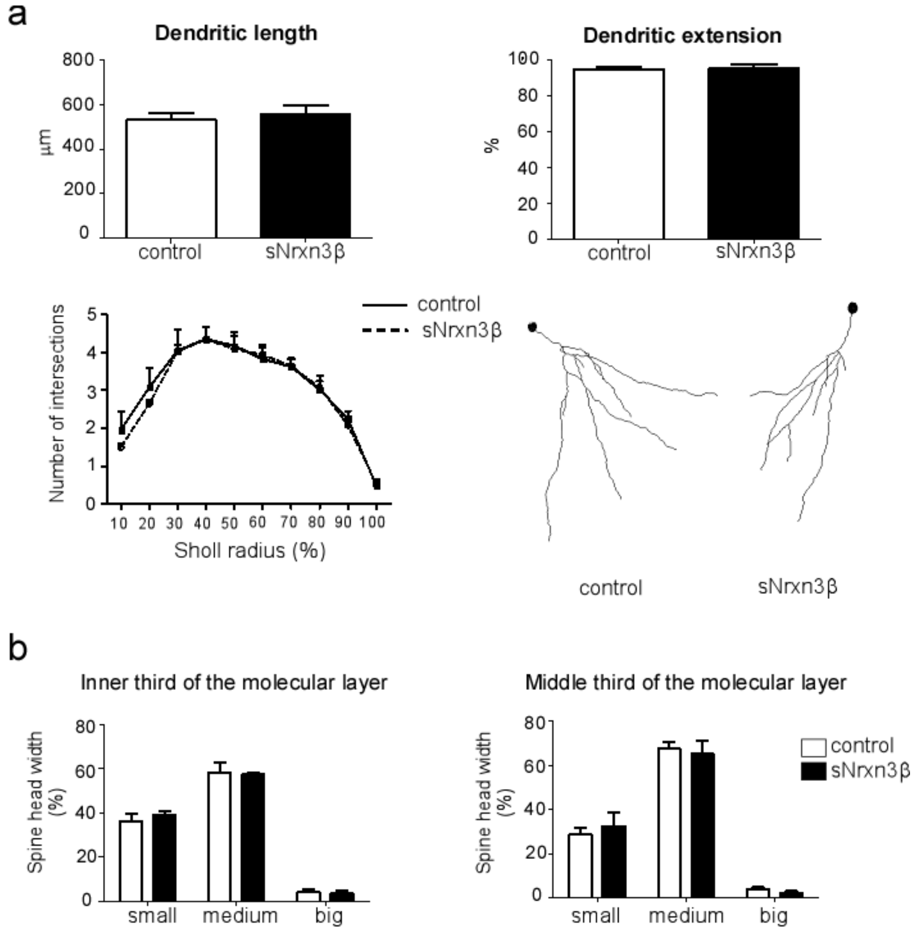

Supplementary Figure S6. **Sholl and spine morphology analysis of NBNs expressing sNrxn3β at 28 d.p.i.** (**a**) Sholl analysis of control and sNrxn3β-overexpressing neurons at 28 d.p.i. Right panel represents the dendritic length (P=0.672) and left panel shows the dendritic extension (P=0.286). Bottom panels show the number of intersections per radius. (10%, P=0.893; 20%, P=0.661; 30%, P=0.464; 40%, P=0.777; 50%, P=0.729; 60%, P=0.661; 70%, P=0.655; 80%, P=0.371; 90%, P=0.371; 100%, P= 0.655). A dendritic arborisation scheme for each group is also represented. (**b**) Percentage of spines in control and sNrxn3β-overexpressing neurons in the inner third of the molecular layer (left panel) and in the middle third of the molecular layer (right panel) (inner third: $F(5,21)=76.95$, small, P=1; medium,

P=1; big, P=1; middle third: $F(5, 21)= 59.87$, small, P=1; medium, P=1; big, P=1). Error bars represent s.e.m.

Supplementary Figure S7. Sholl and spine morphology analysis of NBNs growing in an sNrxn3β enriched environment at 28 d.p.i.

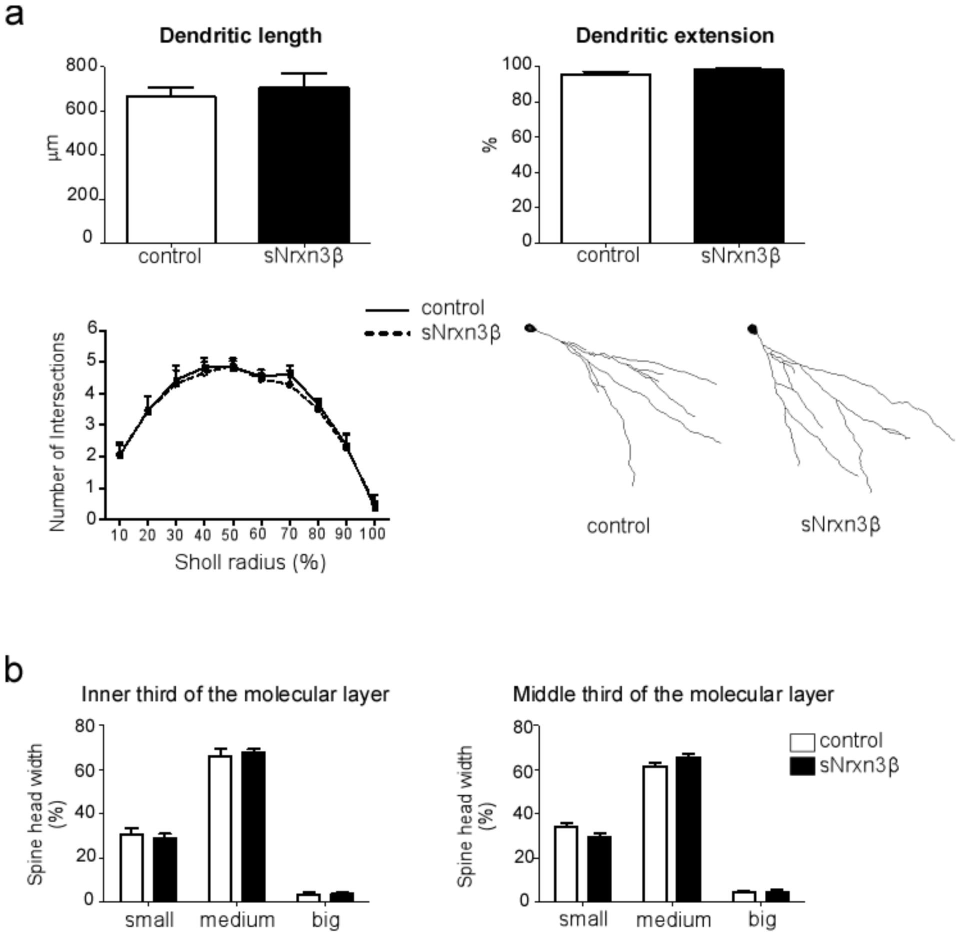

Supplementary Figure S7. Sholl and spine morphology analysis of NBNs growing in an sNrxn3β enriched environment at 28 d.p.i. (a) Sholl analysis of NBNs growing in a sNrxn3β enriched environment 28 d.p.i. Right panel represents the dendritic length (P=0.618) and left panel shows the percentage of maximum dendritic extension (P=0.125). Bottom panels show the number of intersections per radius, together with the dendritic arborisation scheme for each group. No significant differences were found for any of the segments analyzed (10%, P=0.968; 20%, P=0.977; 30%, P=0.835; 40%, P=0.717; 50%, P=0.862; 60%, P=0.734; 70%, P=0.527 80%, P=0.576; 90%, P=0.894; 100%, P= 0.646). **(b)** Percentage of spines of NBNs growing in a sNrxn3β enriched in the inner third (left panel) and middle third of the molecular layer (right panel) ($F(21,5)=64.39$, inner third: small, P=01; medium, P=1;

big, P=1; middle third: F(21,5)=384.12, small, P=1.; medium, P=0.1; big, P=0.1). Error bars represent s.e.m.

Supplementary Figure S8. Alignement of Neurexin 1, 2 and 3 isoforms.

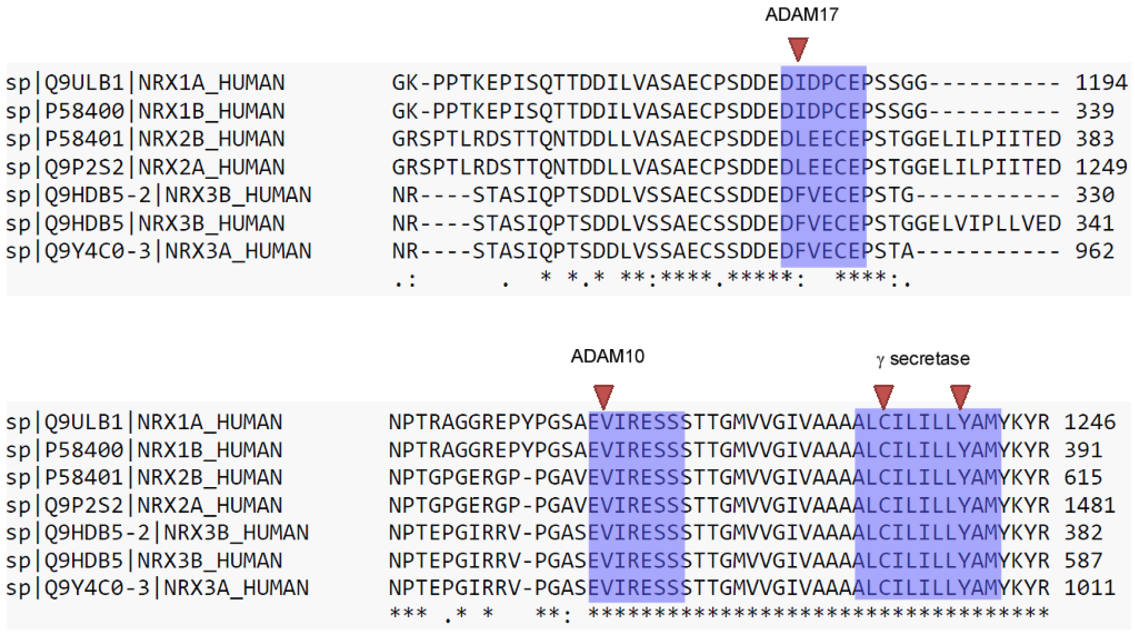

Supplementary Figure S8. Alignement of Neurexin 1, 2 and 3 isoforms. Neurexin 3β cleavage sites are highly conserved among different human neurexin isoforms. Nrxn 1, 2 and 3 isoform sequences were aligned in order to compare the sheddase and γ-secretase cleavage sites using ClustalW at EMBL-EBI (http://www.ebi.ac.uk/Tools/msa/clustalw2/). The ADAM17, ADAM10 and γ-secretase cleavage sites were conserved among different isoforms.

Supplementary Table T1. Primary and secondary antibodies used in the study.

Epitope	Dilution	Reference	Technique	
Mouse anti-FLAG (M2)	1:1000	Sigma-Aldrich, St. Louis, MO, US	WB/IP	**Primary antibodies**
Rabbit anti-C-terminus Neurexin	1:1000	Bot et al., 2013(13)	WB	
Rabbit anti-TACE	1:1000	Calbiochem, San Diego, CA, US	WB	
Rabbit anti-Adam10	1:1000	Calbiochem, San Diego, CA, US	WB	
Rabbit anti-Grin2c	1:1000	Thermoscientific, Rockford, IL, US	WB	
Rabbit anti-beta Actin	1:2000	Sigma St. Louis, MO, US	WB	
Rabbit anti-Rab3a	1:1000	Synaptic Systems, Goettingen, Germany	WB	
Mouse anti-Histidine tag	1:1000	Bio-Rad AbD Serotec, Oxford, UK	WB/IP	
Chicken anti-GFP	1:1000	Biotrend, Cologne, Germany	IHC	
Rabbit anti-MAP2	1:2000	Acris, Hiddenhous, Germany	IHC	
Rabbit anti-RFP	1:1000	Rockland, Gilbertsville, PA, US	IHC	**Secondary antibodies**
Anti chicken Alexa 488	1:250	Biotrend, Cologne, Germany	IHC	
Anti rabbit Alexa 594	1:250	AnaSpec, San Jose, CA, US	IHC	
Anti mouse/rabbit/goat Alexa 680	1:1000	Invitrogen, Carlsbad, CA, US	WB	
Anti mouse/rabbit Alexa 800	1:1000	Invitrogen, Carlsbad, CA, US	WB	
